# Vitamin D3 attenuates SARS‐CoV‐2 nucleocapsid protein‐caused hyperinflammation by inactivating the NLRP3 inflammasome through the VDR‐BRCC3 signaling pathway in vitro and in vivo

**DOI:** 10.1002/mco2.318

**Published:** 2023-06-21

**Authors:** Mingliang Chen, Ying He, Xiaofeng Hu, Xunhu Dong, Zexuan Yan, Qingning Zhao, Jingyuan Li, Dongfang Xiang, Yong Lin, Hongbin Song, Xiuwu Bian

**Affiliations:** ^1^ Institute of Pathology and Southwest Cancer Centre Southwest Hospital Army Medical University Chongqing China; ^2^ Institute of Toxicology School of Military Preventive Medicine Army Medical University Chongqing China; ^3^ Department of Ultrasound Xinqiao Hospital Army Medical University Chongqing China; ^4^ Department of Health Supervision and Surveillance Chinese PLA Center for Disease Control and Prevention Beijing China

**Keywords:** BRCC3, COVID‐19, NLRP3 inflammasome, VDR, Vitamin D3

## Abstract

Severe acute respiratory syndrome coronavirus 2 (SARS‐CoV‐2) infection‐caused coronavirus disease 2019 (COVID‐19) is a global crisis with no satisfactory therapies. Vitamin D3 (VD3) is considered a potential candidate for COVID‐19 treatment; however, little information is available regarding the exact effects of VD3 on SARS‐CoV‐2 infection and the underlying mechanism. Herein, we confirmed that VD3 reduced SARS‐CoV‐2 nucleocapsid (N) protein‐caused hyperinflammation in human bronchial epithelial (HBE) cells. Meanwhile, VD3 inhibited the NOD‐like receptor family pyrin domain containing 3 (NLRP3) inflammasome activation in N protein‐overexpressed HBE (HBE‐N) cells. Notably, the inhibitors of caspase‐1, NLRP3, and *NLRP3* or *caspase‐1* small interference RNA (siRNA) enhanced VD3‐induced NLRP3 inflammasome inactivation, with subsequent suppression of interleukin‐6 (IL6) and IL1β release in HBE‐N cells, which were abolished by the NLRP3 agonist. Moreover, VD3 increased NLRP3 ubiquitination (Ub‐NLRP3) expression and the binding of the VDR with NLRP3, with decreased BRCA1/BRCA2‐containing complex subunit 3 (BRCC3) expression and NLRP3‐BRCC3 association. VD3‐induced Ub‐NLRP3 expression, NLRP3 inflammasome inactivation, and hyperinflammation inhibition were improved by the BRCC3 inhibitor or *BRCC3* siRNA, which were attenuated by the vitamin D receptor (VDR) antagonist or *VDR* siRNA in HBE‐N cells. Finally, the results of the in vivo study in AAV‐Lung‐enhanced green fluorescent protein‐N‐infected lungs were consistent with the findings of the in vitro experiment. In conclusion, VD3 attenuated N protein‐caused hyperinflammation by inactivating the NLRP3 inflammasome partially through the VDR‐BRCC3 signaling pathway.

## Graphical abstract

At present, there is no satisfactory therapy for patients with serious coronavirus disease 2019 (COVID‐19), resulting in severe health problems. Herein, we found that Vitamin D3 (VD3) attenuated‐nucleocapsid (N)protein‐caused hyperinflammation by inactivating the NOD‐like receptor family pyrin domain containing 3 (NLRP3) inflammasome via inducing NLRP3 ubiquitination, and the NLRP3 ubiquitination in part was mediated through the vitamin D receptor‐BRCA1/BRCA2‐containing complex subunit 3（VDR‐BRCC3）  signaling pathway. These findings indicate that VD3 is a feasible treatment option for COVID‐19 and provide new biological evidence for clinical applications of VD3 in COVID‐19 therapy.

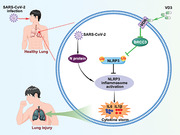

## 1 INTRODUCTION

Severe acute respiratory syndrome coronavirus 2 (SARS‐CoV‐2) infection causes coronavirus disease 2019 (COVID‐19), a costly and persistent global crisis. COVID‐19 is characterized by severe inflammatory responses in the respiratory system.^1^ The excessive release of proinflammatory cytokines forming the so‐called “cytokine storm”, leads to acute respiratory distress syndrome that is the common cause of mortality in COVID‐19 patients.^2^ Pharmaceutical and dietary strategies have targeted these disorders to control COVID‐19, and many natural nutrients with excellent pharmacological properties are good candidates for the treatment and prevention of COVID‐19.^3^, ^4^ Vitamin D3 (VD3), a pro‐hormone synthesized in the skin, that exerts excellent health‐protective effects, is considered a potential treatment option for several virus acute respiratory infections, including COVID‐19.^5^, ^6^, ^7^ A low level of VD3 is positively related to an increased risk of SARS‐CoV‐2 infection and improving VD3 status is potentially beneficial for managing this condition.^8^ However, the exact effects of VD3 on SARS‐CoV‐2 infection or the underlying mechanism are currently unknown.

NOD‐like receptor family pyrin domain containing 3 (NLRP3) inflammasome is the most widely studied and best validated biological target in innate immunity. The NLRP3 inflammasome plays a crucial role in regulating innate immune and inflammatory responses by inducing the secretion of interleukin‐1 (IL‐1) β, which is involved in the pathogenesis of diverse inflammatory diseases, including COVID‐19.^9^, ^10^ It has been demonstrated that the COVID‐19 severity is closely correlated with SARS‐CoV‐2 infection‐induced NLRP3 activation in patients.^11^, ^12^ The lethality of COVID‐19 can be increased by age‐induced overactivation of the NLRP3 inflammasome in elderly patients.^13^ In 2021, Pan et al.^14^ has confirmed that SARS‐CoV‐2 nucleocapsid (N) protein activates the NLRP3 inflammasome thereby inducing excessive inflammatory responses, which are blocked by NLRP3 inhibitors. In 2022, Zeng et al.^15^ also reports that the NLRP3 inflammasome blocking attenuates COVID‐19‐related proinflammatory cytokines release in cells and mice, indicating that targeting the NLRP3 inflammasome would be a potential target for severe COVID‐19 therapy. Moreover, VD3 has been found to inhibit the activation of NLRP3 inflammasome thereby attenuating periodontitis and particulate matter‐caused inflammation.^16^, ^17^ Our recent work also confirmed that VD3 ameliorates nitrogen mustard (NM)‐induced cutaneous inflammation through inhibiting the NLRP3 inflammasome activation.^18^ These published data indicate that the NLRP3 inflammasome is required for the protective effects of VD3. Therefore, we explored whether VD3 could inhibit SARS‐CoV‐2 infection‐induced hyperinflammation by inactivating the NLRP3 inflammasome in our present study.

Recently, deubiquitination has been found to be critical for NLRP3 inflammasome activation.^19^ BRCA1/BRCA2‐containing complex subunit 3 (BRCC3) is a JAMM domain‐containing Zn^2+^ metalloprotease deubiquitinase (DUB), which has been identified as an endogenous DUB regulating NLRP3 through screening a DUBs expression library.^20^ BRCC3 regulates the NLRP3 inflammasome activation by promoting its deubiquitination.^20^ Furthermore, vitamin D receptor (VDR) can physically bind with NLRP3 thereby blocking the association of NLRP3 with BRCC3, subsequently decreasing BRCC3‐mediated NLRP3 deubiquitination, ultimately inhibiting the NLRP3 inflammasome activation.^21^ Reportedly, the beneficial effects of VD3 on several inflammatory diseases such as lupus nephritis and sepsis are abolished in the absence of VDR.^22^, ^23^, ^24^ Additionally, VD3 also suppresses pulmonary emphysema in a VDR‐dependent manner.^25^ Accordingly, we hypothesized that the VDR‐BRCC3 signaling pathway may also be involved in VD3‐induced inactivation of the NLRP3 inflammasome in response to SARS‐CoV‐2 infection.

As expected, our findings indicated, for the first time, that VD3 attenuated N protein‐induced hyperinflammation by inactivating the NLRP3 inflammasome via the VDR‐BRCC3 signaling pathway in vitro in human bronchial epithelial (HBE) cells and in vivo in C57BL/6J mice, at least partially. These results provide new direct evidence regarding the therapeutic effects of VD3 on COVID‐19 as well as clarifying the underlying mechanisms, which may open novel avenues for designing clinical therapeutic strategies for COVID‐19.

## 2 RESULTS

### 2.1 VD3 prevented N protein‐induced hyperinflammation in HBE cells

**FIGURE 1:**
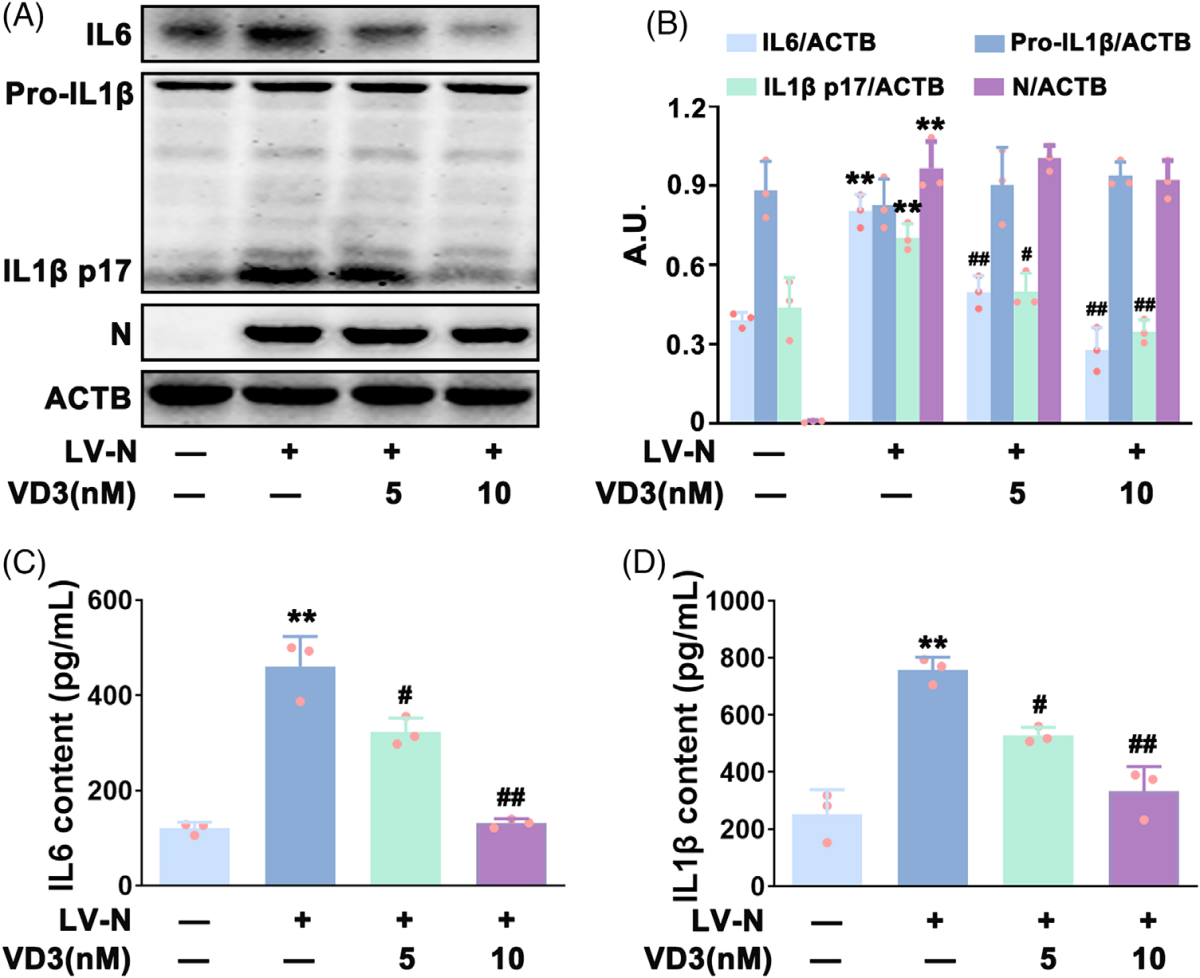
VD3 attenuated N protein induced‐hyperinflammation in HBE cells. (A) Pro‐IL1β, IL1β p17, IL6, and N protein expression were determined via western blot. (B) Bar charts show the quantification of the indicated proteins; arbitrary unit (A.U.). (C) IL6 and (D) IL1β contents in the supernatant fraction were analyzed via ELISA. Values are presented as the means ± standard deviation (*n* = 3); ^**^ *p* < 0.01 versus vehicle‐treated control group, ^#^ *p* < 0.05, ^##^ *p* < 0.01 versus N protein single‐transfected group.

To explore the effect of VD3 on N protein‐induced hyperinflammation, HBE cells were transfected with N protein, and the expression and secretion of IL6 and IL1β p17 were measured by western blot and enzyme‐linked immunosorbent assay (ELISA) kits, respectively. In accordance with previous observations,^14^ HBE cells transfection with N protein (HBE‐N) significantly increased the expression and secretion of IL6 and IL1β p17 (Figure 1A–D). Next, VD3 (5 or 10 nM) was incubated with HBE‐N cells. As expected, VD3 markedly reduced the N protein‐induced IL6 and IL1β p17 expression and secretion in HBE cells (Figure 1A–D). In addition, the secretion of inflammatory factors had not been significantly affected by single‐VD3 (5 and 10 nM) treatment in HBE cells (Figure S1). These results suggested that VD3 attenuated N protein‐triggered hyperinflammation in HBE cells.

### 2.2 VD3 prevented N protein‐induced hyperinflammation by inhibiting the NLRP3 inflammasome in HBE cells

**FIGURE 2:**
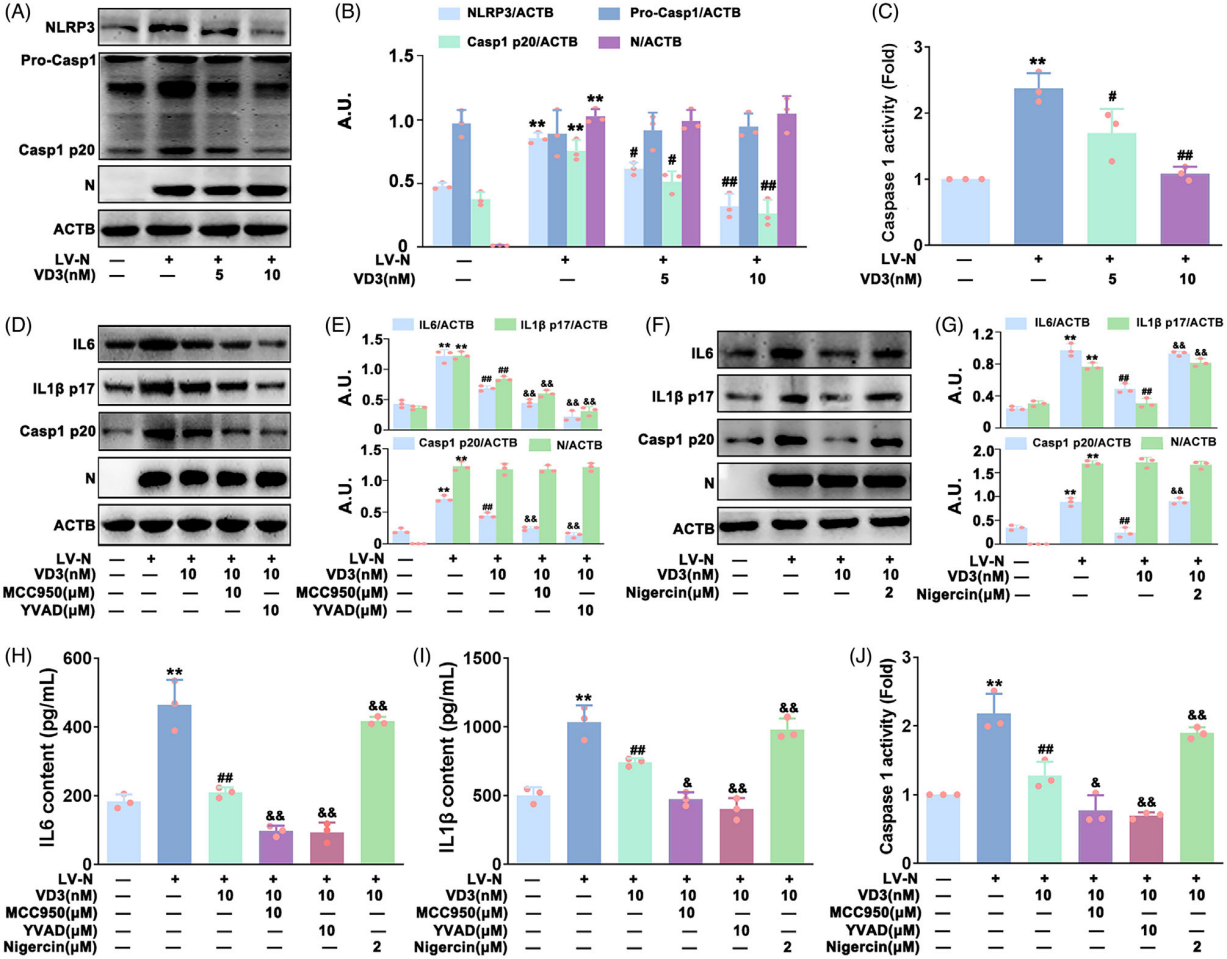
The effect of specific inhibitors and agonists of NLRP3 inflammasome onVD3‐induced attenuation of N protein‐caused hyperinflammation in HBE cells. (A) Western blot analysis of NLRP3, pro‐Casp1, Casp1 p20, and N protein levels. (B) Bar charts show the quantification of the indicated proteins; arbitrary unit (A.U.). (C) Measurement of caspase 1 activity with a specific caspase 1 activity kit. (D) Western blot analysis of IL6, IL1β p17, Casp1 p20, and N protein expression in HBE‐N cells treated with or without YVAD (10 μM) or MCC950 (10 μM). (E) Bar charts show the quantification of the indicated proteins. (F) Western blot analysis of IL6, IL1β p17, Casp1 p20, and N protein expression in HBE‐N cells treated with or without nigericin (2 μM). (G) Bar charts show the quantification of the indicated proteins. ELISA analysis of (H) IL6 and (I) IL1β secretion. (J) Caspase 1 activity was measured using a caspase 1 activity kit. Values are expressed as the means ± standard deviation (*n* = 3).^**^ *p* < 0.01 versus the vehicle‐treated control group; ^#^ *p* < 0.05, ^##^ *p* < 0.01 versus N protein single‐transfected group. ^&^ *p* < 0.05, ^&&^ *p* < 0.01 versus N protein transfection plus VD3‐treated group.

**FIGURE 3:**
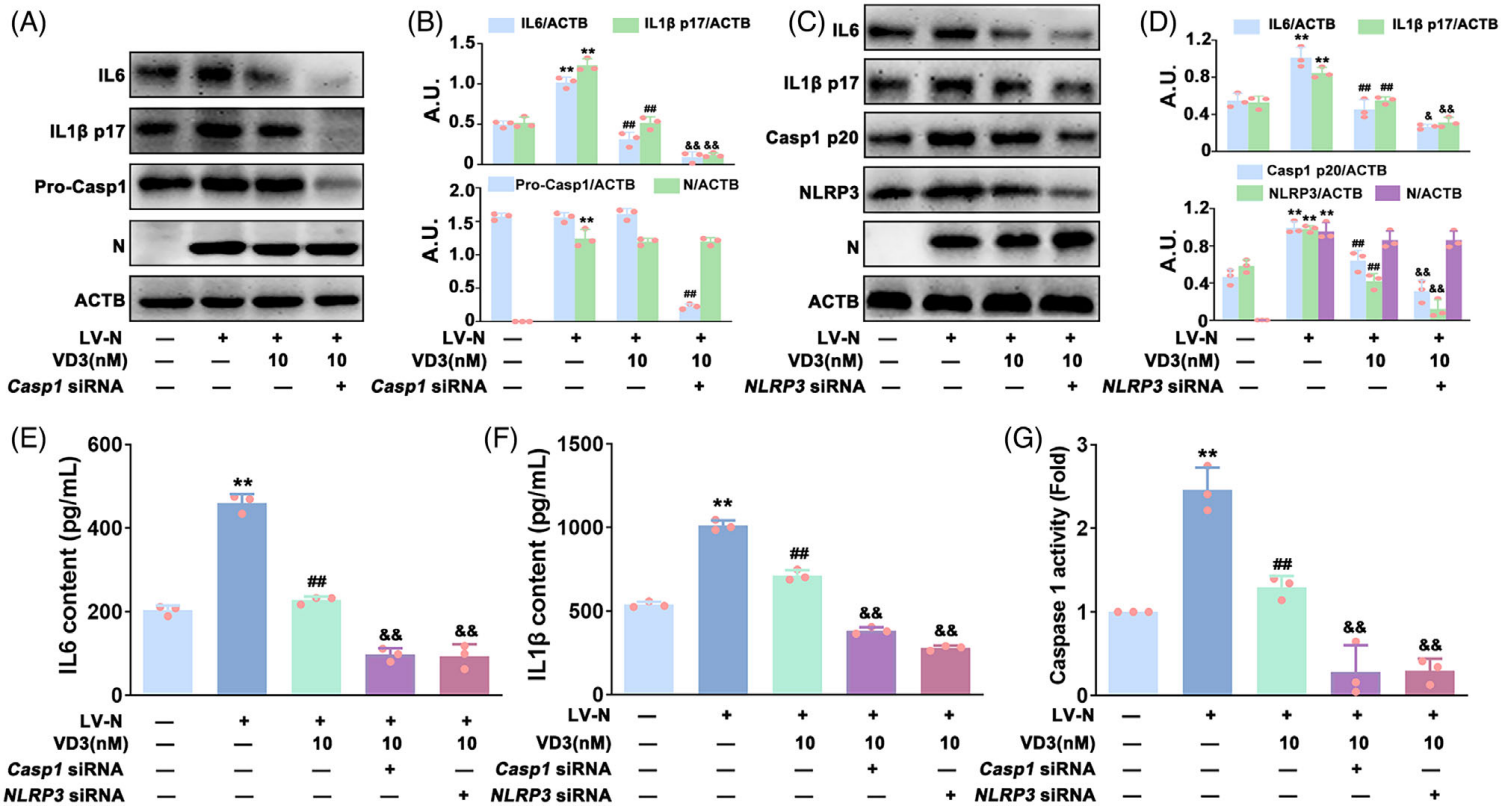
The effect of *NLRP3* siRNA and *Casp1* siRNA on VD3‐induced attenuation of N protein‐caused hyperinflammation in HBE cells. (A) and (C) The level of IL6, IL1β p17, pro‐Casp1, Casp1 p20, NLRP3, and N protein was detected via western blot. (B, D) The bar graphs showing the quantification of the indicated proteins; arbitrary unit (A.U.). (E) IL6 and (F) IL1β contents were detected by ELISA. (G) Caspase 1 activity was measured using a caspase 1 activity kit. Values are expressed as the means ± standard deviation (*n* = 3). ^**^ *p* < 0.01 versus the vehicle‐treated control group; ^##^ *p* < 0.01 versus N protein single‐transfected group. ^&^ *p* < 0.05, ^&&^ *p* < 0.01 versus N protein transfection plus VD3‐treated group.

NLRP3 inflammasome is intensively involved in N protein‐caused hyperinflammation.^12^ Accordingly, the potential involvement of NLRP3 inflammasome in VD3's protective effect on N protein‐induced hyperinflammation was investigated in HBE cells. Western blot and caspase 1 activity detection kit analysis found that pro‐caspase 1 (pro‐casp1) expression was not affected by N protein, but NLRP3 and caspase 1 p20 (casp1 p20) expression and caspase 1 activity were markedly upregulated by N protein in HBE cells, which were dose‐dependently ameliorated by VD3 (Figure 2A–C). Moreover, inhibitors of NLRP3 (MCC950) and caspase‐1 (zYVAD‐fmk, YVAD) were used to further determine the possible role of the NLRP3 inflammasome. As indicated in Figure 2D–J, VD3‐induced decrease of NLRP3 and Casp1 p20 expression, IL1β p17 and IL6 expression and secretion, and caspase 1 activation was markedly enhanced by MCC950 (10 μM) or YVAD (10 μM) in HBE‐N cells. However, these effects were notably abolished by nigericin (a widely used NLRP3 agonist, 2 μM) (Figure 2F–J). Meanwhile, *caspase‐1 (Casp1)* or *NLRP3* small interference RNA (siRNA) also improved VD3‐induced inhibition of the expression of NLRP3, Casp1 p20, IL1β p17, and IL6 expression and release, and caspase 1 activity in HBE‐N cells (Figure 3A–G and Figure S2A,B). These results clearly indicated that VD3 attenuated N protein‐triggered hyperinflammation in an NLRP3‐inflammasome‐dependent manner in HBE cells.

### 2.3 VD3 suppressed N protein‐induced NLRP3 inflammasome activation via the VDR‐BRCC3 signaling pathway in HBE cells

**FIGURE 4:**
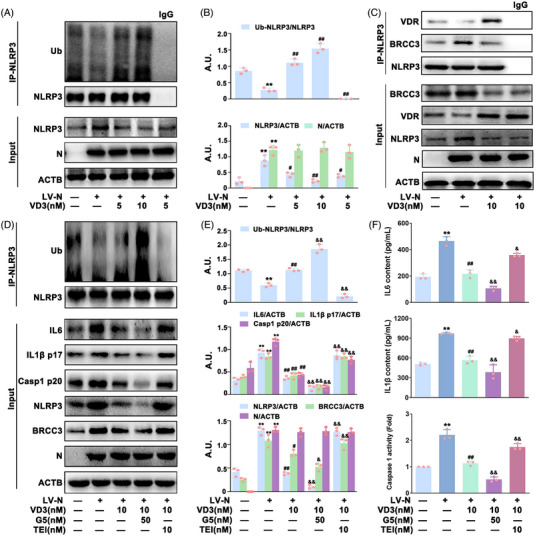
The effect of specific inhibitors of VDR and BRCC3 on VD3‐induced NLRP3 inflammasome inactivation in HBE‐N cells. (A) Cell lysates were immunoprecipitated with anti‐NLRP3 antibody or anti‐rabbit IgG and then immunoblotted with the indicated antibodies; ubiquitination (Ub). (B) The bar graphs showing the quantification of the indicated proteins; arbitrary unit (A.U.). (C) Cell lysates were immunoprecipitated with anti‐NLRP3 antibody or anti‐rabbit IgG and then immunoblotted with the indicated antibodies. (D) Cell lysates of HBE‐N cells treated with or without G5 (50 nM) or TEI (10 nM) were immunoprecipitated with anti‐NLRP3 antibody and then immunoblotted with the indicated antibodies. (E) The bar graphs showing the quantification of the indicated proteins. (F) IL6 contents (top), IL1β contents (middle), and caspase 1 activity (bottom) were measured by ELISA kits or a specific caspase 1 activity assay kit, respectively. Values are expressed as the means ± standard deviation (n = 3)^**^ *p* < 0.01 versus the vehicle‐treated control group; ^#^ *p* < 0.05, ^##^ *p* < 0.01 versus N protein single‐transfected group. ^&^ *p* < 0.05, ^&&^ *p* < 0.01 versus N protein transfection plus VD3‐treated group.

**FIGURE 5:**
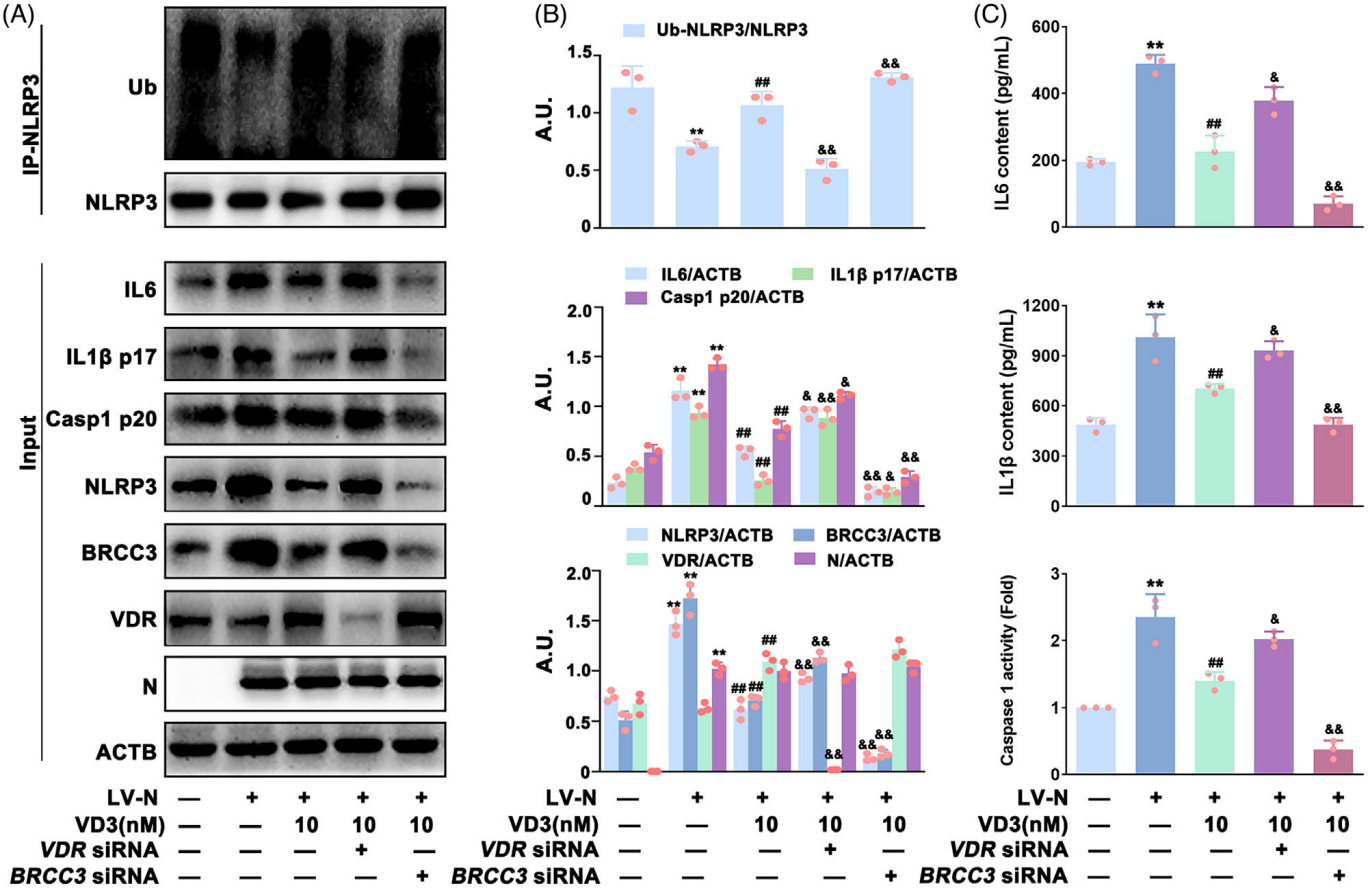
The effect of *VDR* siRNA and *BRCC3* siRNA on VD3‐induced NLRP3 inflammasome inactivation in HBE‐N cells. (A) Cells were collected and lysed and subjected to immunoprecipitated with anti‐NLRP3 antibody and then immunoblotted with the indicated antibodies; ubiquitination (Ub). (B) The bar graphs showing the quantification of the indicated proteins; arbitrary unit (A.U.). (C) IL6 contents (top), IL1β contents (middle), and caspase 1 activity (bottom) were measured by ELISA kits or a specific caspase 1 activity assay kit, respectively. Values are expressed as the means ± standard deviation (n = 3). ^**^ *p* < 0.01 versus the vehicle‐treated control group; ^##^ *p* < 0.01 versus N protein single‐transfected group. ^&^ *p* < 0.05, ^&&^ *p* < 0.01 versus N protein transfection plus VD3‐treated group.

Many studies have demonstrated that ubiquitination is indispensable for controlling inflammasome activation.^26^, ^27^ Therefore, we monitored the ubiquitination of NLRP3 (Ub‐NLRP3) in VD3‐treated HBE‐N cells by immunoprecipitation (IP) of NLRP3 from cells using an anti‐NLRP3 antibody, thereby immunoblotting with anti‐ubiquitin antibody. As indicated in Figure 4A,B, VD3 dose‐dependently increased Ub‐NLRP3 levels in HBE‐N cells. BRCC3 (a DUB) has been found to play a crucial role in controlling Ub‐NLRP3 level and NLRP3 inflammasome activation, which is closely regulated by VDR, the receptor of VD3.^21^ Accordingly, the possible role of the VDR‐BRCC3 signaling pathway in VD3‐induced inhibition of N protein‐caused NLRP3 inflammasome inactivation in HBE cells was investigated. Co‐IP was used to detect the combination of NLRP3 with BRCC3 or VDR. As expected, VD3 significantly increased VDR expression, and the binding of VDR to NLRP3, whereas it decreased the expression of BRCC3 and NLRP3‐BRCC3 association (Figure 4C). Meanwhile, qPCR analysis showed that VD3 had no significant effect on *NLRP3* or *BRCC3* mRNA expression, indicating that *NLRP3* and *BRCC3* were not VDR‐responsive genes at the transcriptional level (Figure S3). Furthermore, inhibitors including G5 (a small‐molecule inhibitor of DUB) and TEI‐9647 (TEI, an antagonist of VDR) were used for further exploration. As shown in Figure 4D–F, VD3‐induced Ub‐NLRP3 was significantly enhanced by G5 (50 nM) followed by a further decrease of NLRP3 and Casp1 p20 expression, IL1β p17 and IL6 expression and secretion, and caspase 1 activity in HBE‐N cells. Meanwhile, TEI (10 nM) treatment notably abolished VD3's effect on the expression of BRCC3, Ub‐NLRP3, NLRP3, and Casp1 p20, the expression and release of IL1β p17 and IL6, and caspase 1 activity (Figure 4D–F). In addition, *BRCC3* or *VDR* siRNA was also applied. As shown in Figure 5A–C and Figure S2C,D, in the presence of *BRCC3* siRNA, VD3‐induced increase of Ub‐NLRP3 level, inhibition of NLRP3 inflammasome activation, and hyperinflammation were markedly enhanced, which were notably abolished by *VDR* siRNA in HBE‐N cells. Our collective data supported the requirement of the VDR‐BRCC3 signaling pathway for VD3‐induced inactivation of the NLRP3 inflammasome and subsequent inhibition of hyperinflammation in HBE‐N cells.

### 2.4 VD3 attenuated N protein‐induced hyperinflammation by inactivating the NLRP3 inflammasome through the VDR‐BRCC3 signaling pathway in vivo

**FIGURE 6:**
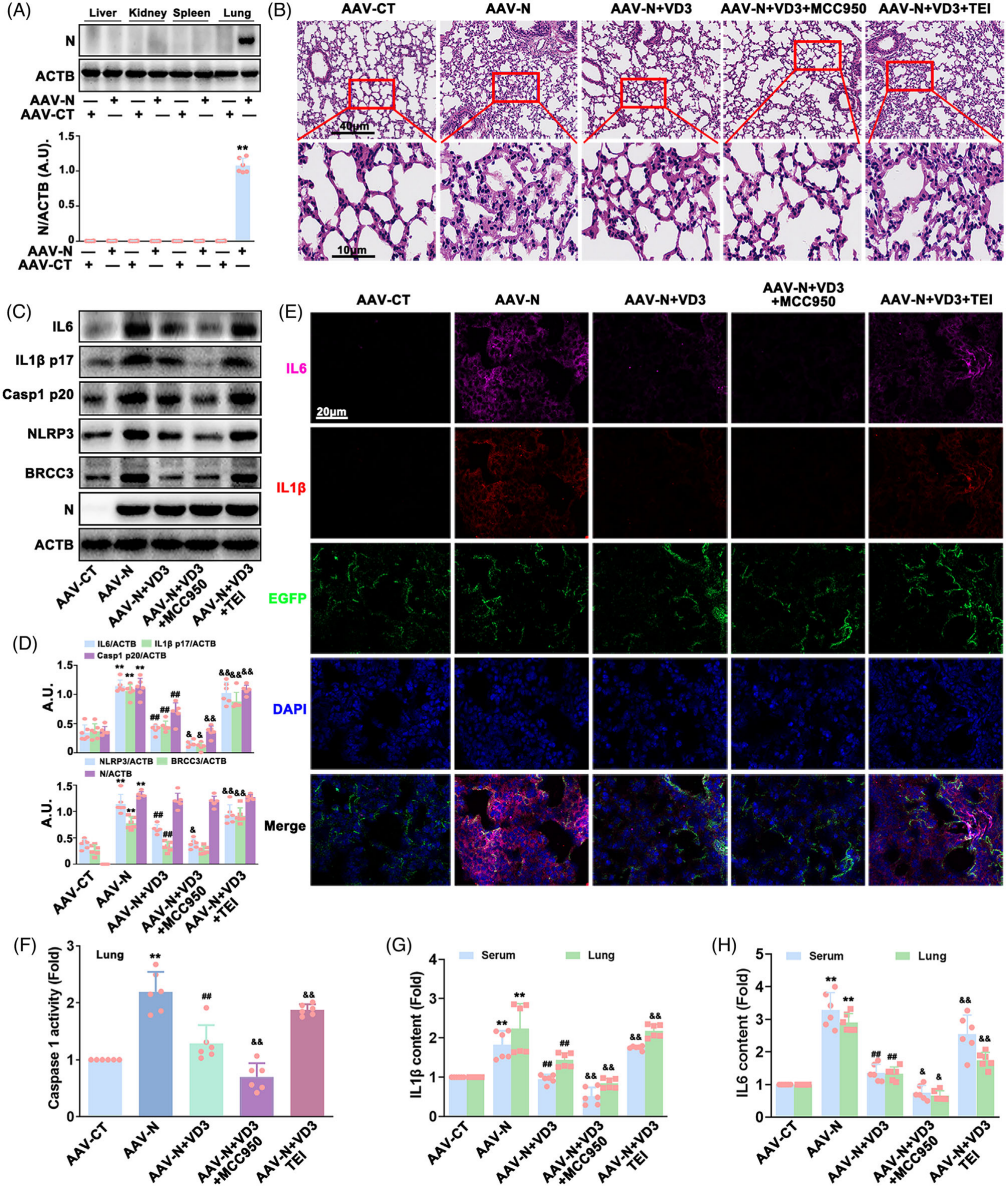
VD3 suppressed SARS‐CoV‐2 N protein‐induced NLRP3 inflammasome activation through the VDR‐BRCC3 signaling pathway in vivo. Mice were sacrificed at 21 days followed by AAV‐N infection. (A) Liver, kidney, spleen, and lung tissues were collected and lysed, the expression of N protein was detected by western blot and the bar graph shows the quantification of N protein; arbitrary unit (A.U.). (B) HE staining was performed to analyze injured lungs. (C) Western blot analysis of IL6, IL1β p17, Casp1 p20, NLRP3, N protein, and BRCC3 expression. (D) Bar graphs show the quantification of the indicated proteins. (E) The expression of IL6 and IL1β was measured by immunofluorescence analysis of the lungs. (F) Assay of caspase 1 activity using a specific kit following the manufacturer's instructions in lungs from AAV‐N‐infected mice. (G) IL1β and (H) IL6 contents in lungs and serum from AAV‐N‐infected mice were determined by ELISA kits, respectively. Values are expressed as the means ± standard deviation (*n* = 6). ^**^ *p* < 0.01 versus the vehicle‐treated control group; ^##^ *p* < 0.01 versus AAV‐N infected group. ^&^ *p* < 0.05, ^&&^ *p* < 0.01 versus AAV‐N infection plus VD3‐treated group.

To ascertain whether VD3‐mediated suppression of N protein‐induced hyperinflammation involves a similar mechanism in vivo, AAV‐Lung‐enhanced green fluorescent protein (EGFP)‐N (AAV‐N) or AAV‐Lung‐EGFP (AAV‐CT) was injected to the tail veins of C57BL/6J mice to establish an N protein‐induced lung injury model, in which the EGFP was used as a marker of AAV to easily detect the transfection efficiency of AAV in lung. Depending on Hematoxylin‐Eosin (HE) staining, western blot, and immunofluorescence analysis, we found that N protein was only expressed in lung tissues and caused lung injury, as evidenced by increasingly infiltrated inflammatory cells, thickened alveolar walls and the destruction of pulmonary alveoli, which were significantly attenuated by VD3 treatment (Figure 6A–D). VD3 induced a significant decrease of BRCC3, NLRP3, Casp1 p20, IL1β p17, and IL6 expression, and caspase 1 activation in the lungs of mice carrying AAV‐N as well as decreasing the release of IL1β and IL6 in serum and lungs (Figure 6C–H). Moreover, MCC950 enhanced VD3‐induced improvement of lung injury, inhibition of NLRP3 inflammasome activation, and hyperinflammation in AAV‐N‐infected mice. In contrast, the protective effects of VD3 on N protein‐caused lung injury, NLRP3 inflammasome activation, and hyperinflammation were abolished by TEI treatment (Figure 6B–H). These data indicated that the VDR‐BRCC3 signaling pathway plays a critical role in VD3‐induced NLRP3 inflammasome inhibition and in the subsequent attenuation of N protein‐caused hyperinflammation in vivo.

## 3 DISCUSSION

At present, there is no satisfactory therapy for patients with serious COVID‐19, resulting in severe health problems.^28^, ^29^ Hence, the development of specific and effective strategies for preventing and treating SARS‐CoV‐2 infection requires urgent action. Many studies have demonstrated that VD3 inhibits the development of virus‐induced respiratory tract infection, especially influenza.^6^, ^30^, ^31^, ^32^ In 2021, a meta‐analysis of 14 studies provides strong evidence that the risk of individuals with sufficient VD3 levels to acquire COVID‐19 is about 80% lower than those with VD3 deficiency, suggesting that VD3 supplementation should be an effective strategy to reduce the risk and severity of COVID‐19.^8^ However, the exact effects of VD3 on SARS‐CoV‐2 infection or the underlying mechanism are currently unknown. Our present study found that VD3 significantly ameliorated N protein‐induced hyperinflammation. This report provided the first direct evidence to demonstrate the efficiency of VD3 supplementation in COVID‐19 therapy. Despite the standard care, many adjunctive drugs have been investigated in clinical trials, including chloroquine, remdesivir, corticosteroids, and umifenovir for COVID‐19 therapy.^4^ Recently, Wu et al. identifies that traditional Chinese medicine and Western medicine combination (TCM‐WM) has better performance in terms of both the progression and outcome of severe COVID‐19 patients, compared to single Western medicine treatment, and they recommend timely TCM‐WM to COVID‐19 therapy.^33^ Our findings are important complements to the previous works, indicating that VD3 is also a feasible adjuvant therapy for COVID‐19.

Accumulating evidence indicates that the NLRP3 inflammasome plays a crucial role in host defense against viruses, while its aberrant activation leads to pathological tissue damage in infections, particularly those caused by SARS‐CoV‐2.^9^, ^34^ SARS‐CoV E protein activates the NLRP3 inflammasome through increasing calcium ions influx and K^+^ efflux and SARS‐CoV 3a could increase the level of mitochondrial reactive oxygen species (mtROS), thereby activating the NLRP3 inflammasome.^35^, ^36^ Recently, it has been found that N protein can interact with NLRP3 protein to facilitate NLRP3 inflammasome assembly and activation. MCC950 and YVAD treatments notably decrease N protein‐caused lung injury and cytokine production.^14^ Zeng et al.^15^ indicates that SARS‐CoV‐2 infection boosts the NLRP3 inflammasome activation accompanied by IL1β and IL18 overproduction, thereby aggravating lung immunopathology in mice, which are abolished by NLRP3 inhibition or knockout. These results clearly support the therapeutic benefits of COVID‐19 in inhibiting SARS‐CoV‐2‐induced NLRP3 inflammasome activation. Moreover, NLRP3 inhibitors have also been tested in a few clinical trials for COVID‐19 such as melatonin (TrialTroveID‐375830), colchicine (TrialTroveID‐381747), and cyclosporine A (TrialTroveID‐383935) and the outcomes of the completed trials are encouraging.^10^ In addition to the clinically applied NLRP3 inflammasome inhibitors in COVID‐19, other potentially effective ones deserve more attention, especially natural nutrients.^10^ For the first time, the present study showed that VD3 could attenuate N protein‐caused hyperinflammation via inactivating the NLRP3 inflammasome in HBE cells and in C57BL/6J mice. These protective effects of VD3 were significantly enhanced by inhibitors or siRNAs of NLRP3 or caspase 1 in N protein‐transfected HBE cells or mice. In contrast, nigericin significantly abolished VD3‐induced inhibition of NLRP3 activation and hyperinflammation in HBE‐N cells. This work provides new insights into the mechanism behind the effectiveness of VD3 in COVID‐19 treatment, in which the NLRP3 inflammasome may play an important role. Additionally, Pan et al. found that NLRP3 expression was not influenced by N protein in HEK293T cells (co‐transfected with Flag‐N plus HA‐NLRP3),^14^ which was different from our results. More recently, it has been found that N protein specifically binds Gasdermin D (GSDM) and protects GSDMD from oligomerization thereby reducing IL1β secretion in monocytes,^37^ which are different from our and Pan's findings that IL1β secretion was notably increased by N protein. The possible reasons might be: 1) N protein has different effects on even the same targeted molecules in different cell types; 2) Our present study detected the endogenous NLRP3 expression, whereas Pan et al. investigated the transfected‐HA‐NLRP3 expression. However, the exact underlying mechanisms accounting for the difference are unknown and need further studies.

Furthermore, the potential mechanisms of VD3‐induced inactivation of the NLRP3 inflammasome were also explored. Increasing evidence has confirmed that the deubiquitination of NLRP3 plays an important role in activating the NLRP3 inflammasome. Several DUBs have been identified to regulate NLRP3 inflammasome activation, among which BRCC3 is the most studied one. It has been demonstrated that BRCC3 promotes NLRP3 deubiquitination by directly binding to NLRP3, thereby favoring NLRP3 oligomerization and activation. These effects are notably abolished in the presence of G5.^20^, ^38^ A later study revealed that BRCC3 deficiency attenuates NLRP3‐associated inflammatory diseases such as peritonitis and sepsis.^39^ The published data clearly suggest that BRCC3 is a positive regulator for activating the NLRP3 inflammasome and that targeting BRCC3 should be a potentially effective strategy for the treatment of NLRP3‐associated inflammatory disorders. More recently, Rao et al.^21^ shows that VDR is able to bind with NLRP3, thereby blocking the association of NLRP3 with BRCC3, which in turn inhibits BRCC3‐mediated NLRP3 deubiquitination. VDR deficiency leads to an increase in BRCC3 expression and NLRP3 deubiquitination, followed by NLRP3 inflammasome activation.^21^ They also find that VD3 attenuates nigericin‐induced NLRP3 inflammasome activation by inhibiting BRCC3‐mediated NLRP3 deubiquitination in a VDR‐dependent manner in bone marrow‐derived macrophages.^21^ Experiments from the current study showed that VD3 increased Ub‐NLRP3, the binding of VDR to NLRP3, and VDR expression; whereas it decreased BRCC3 expression and NLRP3‐BRCC3 association in N protein‐transfected HBE cells and lungs of mice. Notably, G5 or *BRCC3* siRNA improved VD3‐induced NLRP3 deubiquitination as well as the NLRP3 inflammasome inactivation in HBE‐N cells. However, these beneficial effects of VD3 were markedly abolished by TEI or *VDR* siRNA in HBE‐N cells and lungs of AAV‐N‐infected mice. Our data, for the first time, indicated that the VDR‐BRCC3 signaling pathway is vital for VD3‐induced NLRP3 inflammasome inactivation in N protein‐transfected HBE cells and the lungs of mice.

Finally, there are still some limitations of our current study: 1) Our previous work finds that VD3 appears to attenuate NM‐induced NLRP3 inflammasome activation by decreasing mtROS contents through activating the sirtuin 3 (SIRT3)‐manganese superoxide dismutase 2 (SOD2) pathway.^18^ Whether VD3 could attenuate N protein‐induced NLPR3 inflammasome activation through the SIRT3‐SOD2‐mtROS signaling pathway in HBE cells requires further studies. 2) We also failed to identify the role of NLRP3 ubiquitination in N protein‐caused NLRP3 inflammasome activation as well as the exact mechanism by which N protein induced NLRP3 deubiquitination, which remains further exploration. 3) It should be noted that all of our experiments were performed on mice and cells. As such, the efficiency of VD3 in the treatment of COVID‐19 patients needs further research, especially large‐scale, long‐period, randomized controlled trials.

**FIGURE 7:**
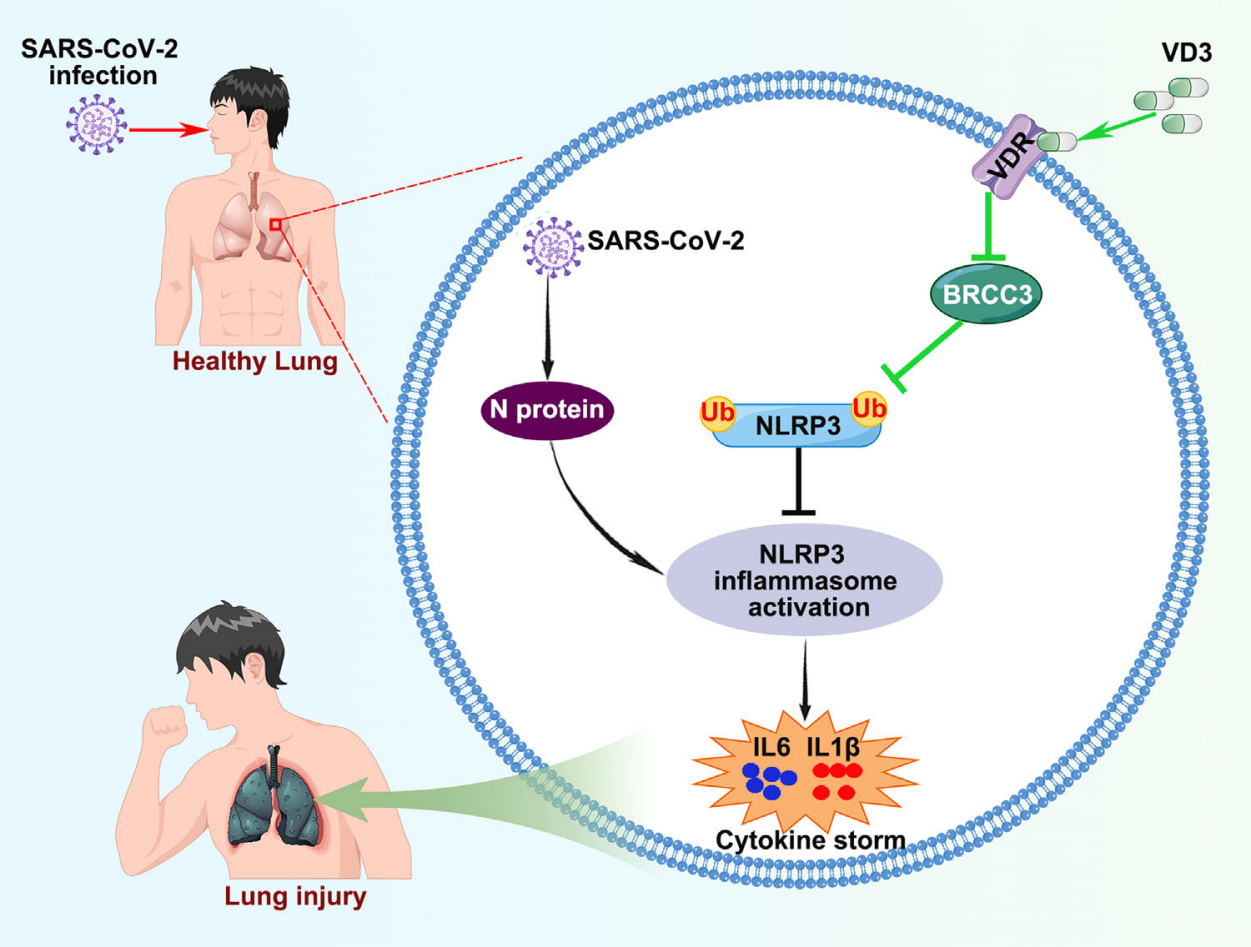
VD3 ameliorates N protein‐induced hyperinflammation by inactivating the NLRP3 inflammasome through the VDR‐BRCC3 signaling pathway.

In general, we have uncovered a novel mechanism whereby VD3 attenuated N protein‐induced hyperinflammation by inactivating the NLRP3 inflammasome partially through the VDR‐BRCC3 signaling pathway (Figure 7). These results provide new insights into the mechanism behind the beneficial effects of VD3 for COVID‐19 therapy, indicating that VD3 is a feasible treatment option for COVID‐19.

## 4 MATERIALS AND METHODS

### 4.1 Cell treatments

The lentiviral vectors encoding the N protein gene (GenBank accession number NC_045512, 75656‐1, LV‐N) were purchased from Gene Chem (Shanghai, China). A stable‐expression N protein HBE cell line was executed by LV‐N transfection according to the manufacturer's instructions. Then, HBE‐N cells were treated with MCC950 (10 μM) or YVAD (10 μM),^40^ TEI (10 nM),^41^ G5 (50 nM),^20^ and nigericin (2 μM)^14^ for 1 h, following by VD3 (10 nM)^18^ treatment for another 24 h. Dimethyl sulfoxide (DMSO) was taken to dissolve the inhibitors and 0.1% DMSO was added to the control group.

### 4.2 Animal treatments

The mice (*n* = 6/group) were injected with 300 μl of AAV‐N (4.4 × 10^10^ vg, 75826‐2) or AAV‐CT (4.4 × 10^10^ vg, CON414) through the tail vein, which was both purchased from Gene Chem (Shanghai, China). After 14 days, VD3 (50 ng/100 μl per mouse, reconstituted in DMSO and diluted in mineral oil) was intraperitoneal injection combined with or without MCC950 (50 mg/kg) or TEI (5 ng/100 μl per mouse).^14^, ^18^ The mice were injected with the same volume of mineral oil as the control group. Mice were sacrificed at 21 days followed by AAV‐N infection. Serum was collected retro‐orbitally, and lung, kidney, liver, and spleen tissues were collected and fixed in 10% formalin for histopathological analysis or snap‐frozen in liquid nitrogen for western blot analysis.

### 4.3 Western blot analysis

Cells and tissues including lung, kidney, spleen, and liver were collected, lysed, and homogenized for protein extraction and subjected to western blotting, as described previously.^42^ Briefly, 10%–15% SDS‐PAGE was used to resolve the protein (40–120 μg) and electroblotted onto polyvinylidene difluoride membranes. Next, 5% skimmed milk was added to block the membranes for 2 h. Thereafter, the membranes were incubated overnight at 4°C with primary antibodies at the following dilutions: VDR (1:1000), BRCC3 (1:1000), ubiquitin (1:1000), N (1:5000), NLRP3 (1:1000), caspase‐1 (1:400), IL‐1β (1:500), IL6 (1:800) and β‐actin (ACTB, 1:1000). After washing with Tris‐buffered saline containing 0.1% Tween 20 (TBST), membranes were incubated with the appropriate secondary antibodies. Following TBST washes, protein bands were visualized with electrogenerated chemiluminescence using the Vilber Fusion FX7 system.

### 4.4 SiRNA assay

*VDR* siRNA (1103; Sense: 5′‐CCUGCUCAGAUCACUGUAUTT‐3′, Antisense: 5′‐AUACAGUGAUCUGAGCAGGTT‐3′), *NLRP3* siRNA (2999; Sense: 5′‐GGACCUCAGUGACAAUUCUTT‐3′, Antisense: 5′‐AGAAUUGUCACUGAGGUCCTT‐3′), C*asp1* siRNA (918; Sense: 5′‐GGUGUGGUUUAAAGAUUCATT‐3′, Antisense: 5′‐UGAAUCUUUAAACCACACCTT‐3′), *BRCC3* siRNA (279; Sense: 5′‐GGACCGAGUAGAAAUUUCUTT‐3′, Antisense: 5′‐AGAAAUUUCUACUCGGUCCTT‐3′) or negative control siRNA were obtained from GenePharma (Shanghai, China). For transfection, 80–100 nmol siRNA combined with 4 μl Lipofectamine RNAiMAX were incubated with HBE or HBE‐N cells in Opti‐MEM^®^ I reduced serum medium (31985070, Gibco) for 5–7 h as described before.^40^ Cells were subsequently washed and incubated with fresh RPMI‐1640 for a further 24 h. Next, cells were harvested for evaluation of target protein expression or incubated with specific reagents. At the end of the incubation period, cells were harvested and subjected to western blot and other analyses.

### 4.5 Assay of NLRP3 ubiquitination

Ub‐NLRP3 was assayed by IP of NLRP3 from cells using an anti‐NLRP3 antibody, followed by immunoblotting with an anti‐ubiquitin antibody as previously reported.^43^ Briefly, cells were lysed with the denaturation buffer and boiled for 10 min, then diluted with 10 volumes of binding buffer. The diluted lysates were immunoprecipitated with the NLRP3 antibody and protein A/G magnetic beads overnight at 4°C with rotation, and the beads were then washed with binding buffer three or four times, followed by incubating with elution buffer for 5 min. Finally, the standard immunoblotting technique was applied to detect the level of Ub‐NLRP3.

### 4.6 Immunofluorescence analysis

At 21 days after AAV‐N injection, the lung tissues were collected, embedded in optimal cutting temperature compound at −20°C, and sectioned (6–8 μm). Then, the expression of IL1β and IL6 was detected by immunofluorescence analysis accordingly to our previously published methods.^42^ Briefly, antibodies of IL1β and IL6 were diluted at 1:300 and 1:200 and were incubated with the sections at 4°C for 12 h, respectively. After three times washing with phosphate‐buffered saline for 5 min, the sections were incubated with Alexa Fluor 555 antibody or Alexa Fluor 647 antibody for 2 h at 20–25°C. The nuclei were stained by 4′,6‐diamidino‐2‐phenylindole (DAPI) for 10 min at room temperature. Then, the sections were mounted on glass slides. Finally, ZEISS LSM900 confocal laser scanning microscope (ZEISS, Germany) was applied to detect IL1β and IL6 expression in the sections.

### 4.7 HE staining and histopathological analysis

At 21 days after the AAV‐N injection, lung tissues were collected and fixed in 10% formalin for histological analysis. Subsequent preparation for paraffin embedding was performed based on routine protocols. Slice thickness was limited to 5 μm for HE staining and microscopic evaluation (Carl Zeiss, Germany) of histopathological features, such as the accumulation of inflammatory cells, diffuse alveolar damage, and thickening of the alveolar epithelium.^2^

## AUTHOR CONTRIBUTIONS

M.‐L.C., Y.H., X.‐F.H, X.‐H.D, H.‐B.S., and X.‐W.B were involved in the study design. M.‐L.C., Y.H., X.‐F.H., Z.‐X.Y., X.‐H.D., Q.‐N.Z, J.‐Y.L., D.‐F.X, and Y.L conducted the experiments and the statistical analyses. M.‐L.C., Y. H., X.‐F.H., X.‐H.D., H.‐B.S., and X.‐W.B. wrote the first draft of the manuscript. All authors contributed to the final version of the manuscript. H.‐B.S. and X.‐W.B had the primary responsibility for the final content. All authors have read and approved the final manuscript.

## CONFLICT OF INTEREST STATEMENT

The authors declare no conflict of interest.

## ETHICS STATEMENT

All animal experiments were carried out in strict accordance with the recommendations in the Guide for the Care and Use of Laboratory Animals by the National Institutes of Health and were approved by the Animal Care and Use Committee of the Army Medical University (AMUWEC20212432, Chongqing, China).

## Supporting information



Supporting Information

## Data Availability

All data and materials are available to the researchers once published.
