# Supplementary material for: Vitamin D3 attenuates SARS‐CoV‐2 nucleocapsid protein‐caused hyperinflammation by inactivating the NLRP3 inflammasome through the VDR‐BRCC3 signaling pathway in vitro and in vivo
Source: MedComm (2020). 2023 Jun 21;4(4):e318. doi: 10.1002/mco2.318 (PMC10285036; doi:10.1002/mco2.318)
Supplement: Supplementary file 1 — Supporting Information [file MCO2-4-e318-s001.docx]

**Supplementary Materials for:**

**Vitamin D3 attenuates SARS-CoV-2 nucleocapsid protein-caused hyperinflammation by inactivating the NLRP3 inflammasome through the VDR-BRCC3 signaling pathway in vitro and in vivo**

Mingliang Chen^1, 2#^, Ying He^3#^, Xiaofeng Hu^4#^, Xunhu Dong^2#^, Zexuan Yan^1^, Qingning Zhao^1^, Jingyuan Li^1^, Dongfang Xiang^1^, Yong Lin^1^, Hongbin Song^4*^, Xiuwu Bian^1*^

^#^These authors contribute equally to this work

^1^Institute of Pathology and Southwest Cancer Centre, Southwest Hospital, Army Medical University, Chongqing, 400038, China.

^2^Institute of Toxicology, School of Military Preventive Medicine, Army Medical University, 30 Gaotanyan Street, Shapingba District, Chongqing 400038, China.

^3^Department of Ultrasound, Xinqiao Hospital, Army Medical University, Chongqing 400037, China.

^4^Chinese PLA Center for Disease Control and Prevention, Beijing, China

***Correspondence**

Hongbin Song, Ph.D., Professor, Director,

Chinese PLA Center for Disease Control and Prevention No.20 Dongdajie Street,Fengtai District, Beijing, 100071,China Email:hongbinsong@263.net

Xiuwu Bian, M.D., Ph.D., Professor, Director,

Institute of Pathology and Southwest Cancer Centre, Southwest Hospital, Army Medical University, Chongqing 400038, China. E-mail: bianxiuwu@263.net

**Running title:** VD3 ameliorates N protein-induced hyperinflammation **1. Materials and methods**

**1.1 Reagents and antibodies**

RPMI-1640 Medium (SH30809.01B) and fetal bovine serum ([SH30370.03](http://www.bioon.com.cn/reagent/show_product.asp?id=1237790)) were purchased from Hyclone Laboratories (Logan, UT, USA). DMSO (D2650) was purchased from Sigma-Aldrich (St. Louis, MO, USA). YVAD (HY-16990), MCC950 (HY-12815a), G5 (HY-100738), TEI (HY-12398) and VD3 (HY-10002/HY-15398) were got from Med Chem Express (New Jersey, USA). DAPI (C1005), human IL1β ELISA kit (PI305), mouse IL1β ELISA kit (PI301), human IL6 ELISA kit (PI330), mouse IL6 ELISA kit (PI326), caspase 1 activity kit (C1102) were obtained from Beyotime Institute of Biotechnology (Shanghai, China). Antibodies were purchased as follows: antibodies against IL1β (66737-1-Ig), IL6 (21865-1-AP), SARS-CoV-2 N protein (67666-1-Ig), VDR (67192-1-Ig) and BRCC3 (15391-1-AP) were obtained from Proteintech (Wuhan, China). anti-IL1β (12703/12242) and anti-ubiquitin (3933/5839) were from Cell Signaling Technology (Danvers, MA, USA); anti-NLRP3 (ab263899) was purchased from Abcam (Cambridge, MA). Caspase 1 antibody (NBP1-45433) was obtained from NOVUS Biologicals (Colorado, USA), whereas antibody against β-actin (ACTB, TA-09) was obtained from Zhongshan Jinqiao Biotechnology Co (Beijing, China). Pierce Crosslink Magnetic immunoprecipitation (IP) /Co-IP Kit (88805), Lipofectamine™ RNAiMAX transfection reagent (13778150), Alexa Fluor® 555 goat anti-mouse IgG (H+L) antibody (A21422) and Alexa Fluor® 647 goat anti-rabbit IgG (H+L) antibody (A32795) were acquired from Invitrogen (Carlsbad, CA, USA).

**1.2 Cell culture**

HBE cell line HBE135-E6E7 (ATCC® CRL-2741™) was purchased from ATCC (Gaithersburg, Maryland, USA) and cultured in Roswell Park Memorial Institute (RPMI)-1640 supplemented with 10% fetal bovine serum in a humidified atmosphere containing 5% CO_2_ at 37℃. The medium was changed at 2-day intervals and cells were re-plated at 80-90% confluence.

**1.3 Animals**

Female C57BL/6J mice approximately 6-week-old weighing 18–24 g were purchased from Hunan SJA Laboratory Animal Co. Ltd. (Hunan, China) and maintained on a standard laboratory diet. All animal experiments were approved by the Animal Care and Use Committee of the Army Medical University (AMUWEC20212432, Chongqing, China).

**1.4 Measurement of IL1β and IL6 contents**

After various treatments, IL1β and IL6 contents in culture supernatant fractions, serum and lungs were determined with the human and mouse IL1β or IL6 ELISA kit following the manufacturer’s instructions, respectively. Absorption at 450 nm was assessed using an Infinite™ M200 Microplate Reader (Tecan Group Ltd.). Results were calculated based on a calibration curve generated with different concentrations of IL1β and IL6 standards.

**1.5 Measurement of caspase 1 activity**

After being subjected to the appropriate treatments, cells and lung tissues were collected, lysed and homogenized for protein extraction. Thereafter, caspase-1 activity was determined with commercial cell lysate assay kits, in keeping with the manufacturer’s instructions. Protein concentrations were assessed with a Bradford protein assay kit (P0006, Beyotime, China). Absorption at 405 nm was measured using an Infinite™ M200 Microplate Reader (Tecan Group Ltd.).

**1.6 Co-IP**

The combination of NLRP3 with BRCC3 or VDR was detected by co-IP as described before ^[1](#_ENREF_1)^. The cells were collected and resuspended in lysis buffer (50 mM Tris-HCl, 5 mM EDTA, 150 mM NaCl, 0.5% (vol/vol) Nonidet-P40, and 10% (vol/vol) glycerol, pH 7.4) supplemented with 1 mM PMSF and complete protease inhibitor cocktail. Cell lysates were immunoprecipitated with the NLRP3 antibody and beads overnight at 4°C with rotation in keeping with the manufacturer’s protocol. The immunocomplexes were washed three times in lysis buffer the following day, resolved by SDS-PAGE, and analyzed by western blotting.

**1.7 qPCR assays.**

Primer Express software (Applied Biosystems, Foster City, CA, USA) was used to design oligonucleotide primers. PCR assays were performed in 96-well optical reaction plates using an ABI 7500HT thermal cycler (Applied Biosystems) as described previously ^[2](#_ENREF_2)^. Briefly, total RNA was extracted from cells using Trizol reagent, and 1 µg of RNA reverse transcribed into cDNA using the primers as follow: NLRP3: Forward: 5’-TGGATGGGTTTGCTGGGAT-3’, Reverse: 5’-CTGCGTGTAGCGACTGTTGAG-3’; BRCC3: Forward: 5’-GTGCAGGCGGTTCATCTTGA-3’, Reverse: 5’-AACTCCCCTATACACAGACCC-3’; glyceraldehyde 3-phosphate dehydrogenase (GAPDH): Forward: 5’-GCAAAGTGGAGATTGTTGCCAT-3’, Reverse: 5’-CCTTGACTGTGCCGTTGAATTT-3’. A 25-µL reaction containing 4 µg of cDNA, 12.5 µL of SYBR Master mix, and 1 µL of each primer was subjected to thermal cycling, involving an initial denaturation step at 95°C for 10 min and 45 cycles of ampliﬁcation (95°C for 15 s, 60°C for 1 min). All ampliﬁcation reactions were performed in triplicate, and the averages of the threshold cycle were used to interpolate curves using 7300 System SDS Software. Results are presented as the ratio of each mRNA compared with the expression level of GAPDH mRNA.

**1.8 Statistical analyses**

Quantitative data are presented as the means ± standard deviation (SD) of three independent experiments. The statistical analysis was conducted with the *t*-test and one-way analysis of variance using SPSS 23.0 statistical software (SPSS Inc., Chicago, IL, USA). A *P*-value < 0.05 was considered statistically significant and the Tukey-Kramer post-hoc test was applied if *P* < 0.05.


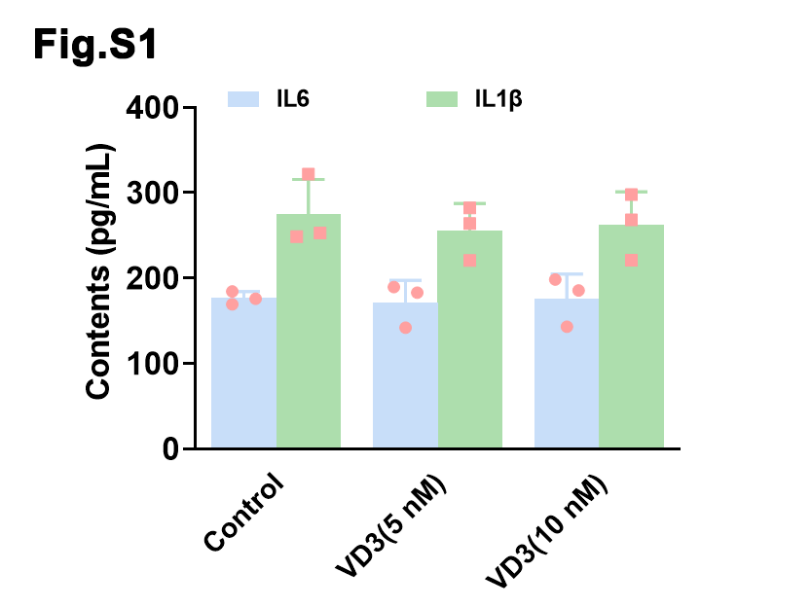


**Figure S1. The effect of VD3 on the secretion of IL6 and IL1β in HBE cells, related to Figure 1.** IL6 and IL1β contents in the supernatant fraction were analyzed via ELISA; Values are presented as the means ± SD (n = 3).


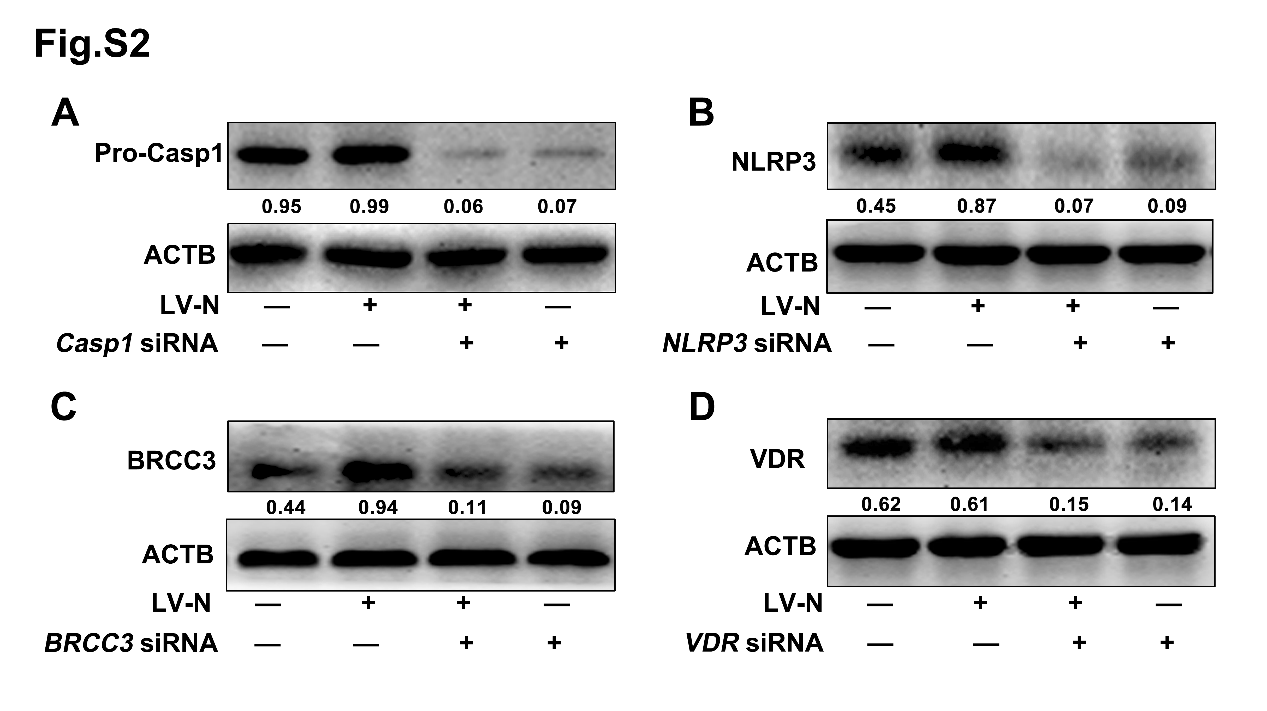


**Figure S2. The knockdown efficiency of siRNAs on the targeted proteins, related to Figure 3 and Figure 5.** The expression of **(A)** pro-Casp1, **(B)** NLRP3, **(C)** BRCC3 and **(D)** VDR was determined by western blot and the representative immunoblots were quantiﬁed by densitometric analysis.


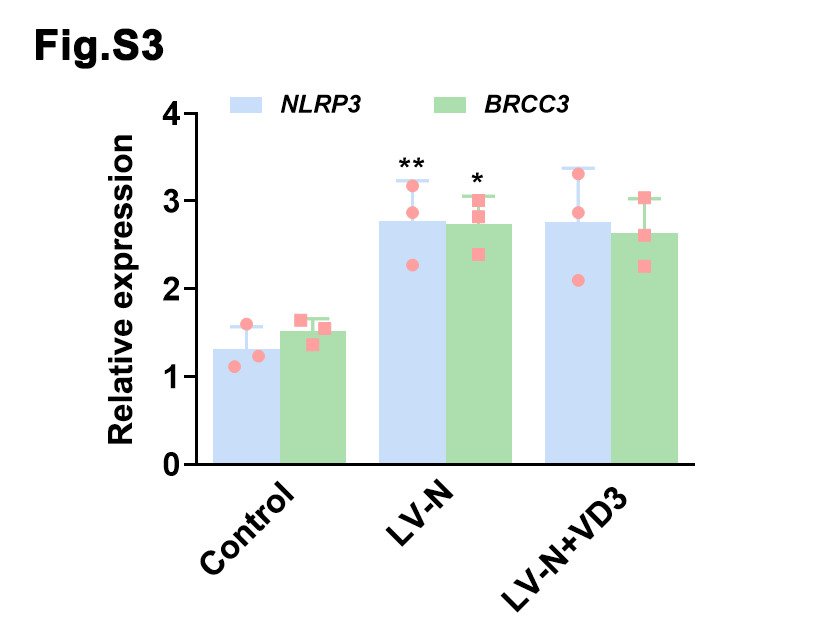


**Figure S3. The effect of VD3 on *NLRP3* and *BRCC3* mRNA expression in HBE-N cells, related to Figure 5.** The expression of *NLRP3* and *BRCC3* mRNA in HBE-N cells treated with or without VD3 (10 nM) was assessed by qPCR assay. Values are expressed as the mean ± SD (*n* = 3); ^*^*p* < 0.05, ^**^*p* < 0.01 versus vehicle-treated control group.

**References**

1. Rao Z, Chen X, Wu J, et al. Vitamin D Receptor Inhibits NLRP3 Activation by Impeding Its BRCC3-Mediated Deubiquitination. *Front Immunol*. 2019;10:2783.

2. Chen, ML, Yi L, Zhang Y, et al*.* Resveratrol Attenuates Trimethylamine-N-Oxide (TMAO)-Induced Atherosclerosis by Regulating TMAO Synthesis and Bile Acid Metabolism via Remodeling of the Gut Microbiota. *mBio.*2016;7(2):e02210-02215.
